# Vision screening in newborns and early childhood

**DOI:** 10.1186/s12887-021-02606-2

**Published:** 2021-09-08

**Authors:** Sophie Jullien

**Affiliations:** Barcelona Institute for Global Health, University of Barcelona, Barcelona, Spain

**Keywords:** Vision screening, Congenital cataract, Retinoblastoma, Amblyopia, Strabismus

## Abstract

We looked at existing recommendations and supporting evidence on the effectiveness of screening for visual disorders in newborns and small infants, and in children between six months and five years of age.

We conducted a literature search up to the 5th of August 2019 by using key terms and manual search in selected sources. We summarized the recommendations and the strength of the recommendations when and as reported by the authors. We summarized the main findings of systematic reviews with the certainty of the evidence as reported on the accuracy of screening tests for detecting visual alterations; the efficacy of treatment for improving visual acuity, school performance, and quality of life; and potential harms derived from vision screening and treating visual alterations.

Although there is little evidence supporting its validity and effectiveness, examining all newborns for congenital cataract and retinoblastoma through the red reflex examination is widely accepted due to the severity of both diseases and the good outcomes reached by early detection and treatment.

Overall, there is a moderate certainty of evidence that visual screening in children between three and five years provides a moderate net benefit, as assessed by the US Preventive Services Task Force: vision screening tests are accurate for detecting amblyopia and its risk factors, and their treatment is associated with visual improvement. There is uncertain evidence on whether vision screening in children under three years of age provides net benefits. Among populations with a low prevalence of vision abnormalities, screening the youngest is associated with an increased rate of false positives, leading to unnecessary additional assessment.

## Background

### Introduction

The World Health Organization (WHO) European Region is developing a new pocket book for primary health care for children and adolescents in Europe. This article is part of a series of reviews, which aim to summarize the existing recommendations and the most recent evidence on preventive interventions applied to children under five years of age to inform the WHO editorial group to make recommendations for health promotion in primary health care. In this article, we looked at existing recommendations and supporting evidence on the effectiveness of screening for visual disorders in newborns and small infants, and in children between six months and five years of age.

### Why is vision screening important in newborns and early childhood?

The aim of vision screening in newborns and early childhood is to detect abnormalities that could lead to severe visual impairment or permanent vision loss if left untreated.

### Context

In newborns and small infants, congenital cataract and retinoblastoma are relatively infrequent but can potentially lead to vision loss and even death in the case of retinoblastoma. The median prevalence of congenital cataracts was estimated at 1.7 per 10,000 children globally and the incidence of retinoblastoma was estimated at 6.0 and 7.1 per 100,000 live births in Northern Europe [1–3]. Early identification of these disorders could lead to early treatment with improved outcomes.

In infancy and preschool age children, one of the leading causes of vision impairment is amblyopia, known as ‘lazy eye’, with an estimated prevalence between 1 and 5% [4, 5]. Amblyopia refers to a decrease in visual acuity from one or both eyes, that arises during the period of visual development and which is not attributed to a structural alteration of the eye or visual pathways. The main risk factors associated with amblyopia include strabismus (ocular misalignment), significant bilateral refractive errors that cause blurred vision (myopia, hyperopia, astigmatism), and anisometropia (asymmetric retractive error). Less common risk factors are vision deprivation caused by media opacity (such as cataracts) or ptosis. Amblyopia is more common in prematurity, low birth weight, and when there is a positive family history, as those are risk factors for developing amblyogenic factors [4, 5]. Amblyopia and its risk factors are unlikely to resolve spontaneously and, if untreated, they can result in not only vision loss, but also in other consequences such as accidents, poor reading ability and suffering from bullying, depression, anxiety, or poor self-esteem [6]. Screening preschool age children for vision impairment could help identify those who may benefit from early interventions to correct or improve vision.

## Key questions

### Newborns and small infants

1. Is screening for visual disorders in newborns and small infants associated with improved outcomes?

### Children between six months and five years of age

2. How accurate are the screening tests for detecting strabismus, refractive errors, anisometropia, and amblyopia in primary care among children between six months and five years of age?
3. What are the potential harms derived from screening children between six months and five years of age for strabismus, refractive errors, anisometropia, and amblyopia?
4. Is treatment of strabismus, refractive errors, media opacity, and amblyopia in children between six months and five years of age effective for improving visual acuity, school performance, and quality of life?
5. What are the potential harms derived from treating strabismus, refractive errors, anisometropia, and amblyopia in children between six months and five years of age?
6. Is population-based screening in children between six months and five years of age effective for improving long-term visual acuity, school performance and quality of life?

## Search methods and selected manuscripts

We described the search methods, data collection and data synthesis in the second paper of this supplement (Sophie Jullien, Gottfried Huss & Ralf Weige. Supporting recommendations for childhood preventive interventions for primary health care: elaboration of evidence synthesis and lessons learnt. BMC Pediatrics. 2021. 10.1186/s12887-021-02638-8).

The search was conducted on the 5th of August 2019, by manual search and by using the search term “vision screening”, “vision test”, “vision disorder”, or “amblyopia”. We included any document that addressed at least one of the key questions. The WHO has developed several strategies and promotional activities to address blindness and vision impairment worldwide [7]. This WHO World report will contain recommendations including comprehensive and integrated eye care with the objective of helping to decrease the burden of eye diseases and vision impairment globally. However, we did not find any specific recommendations on screening measures for children other than eye examination in newborns. We found recommendations and their supporting evidence from the US Preventive Services Task Force (USPSTF) (2017) and the PrevInfad workgroup (Spanish Association of Primary Care Pediatrics) (2016). We looked at the National Institute for Health and Care Excellence (NICE) and Centers for Disease Control and Prevention (CDC) guidelines, as well as the recommendations from the American Academy of Pediatrics (AAP) in collaboration with the American Academy of Ophthalmology (AAO), the Royal College of Paediatrics and Child Health (RCPCH), and the UK National Screening Committee, and we included the brief recommendations that each of them provided.

The supporting evidence document for the USPSTF included a systematic review on screening for amblyopia and its risk factors in children. We searched in the Cochrane library for the identification of other systematic reviews on any topic related to our key questions. By using the search terms cited above, the search returned 99 reviews and two protocols. By screening the titles and abstracts, we included seven systematic reviews that addressed any of the key questions, two on screening and five on treatment of vision impairment. Among the two reviews on screening, one was published in 2017 and looked at tests for detecting strabismus in children between 1 and 6 years of age in the community. The other review assessed vision screening for amblyopia in childhood. Although it was published in 2009, we decided to include it due to the relevance of the topic and the lack of updated Cochrane review addressing this question.

All the included manuscripts for revision in this article are displayed in Table 1.

**Table 1:** Included manuscripts for revision

Sources	Final selected manuscripts
**Recommendations**	
**WHO**	None
**USPSTF**	• 2017 recommendations [4] • Jonas 2017 – Evidence support and systematic review [6]
**PrevInfad**	• 2016 recommendations and supporting evidence [5]
**CDC**	• Guidelines for children’s vision health [8]
**NICE**	• NICE clinical guidelines 2015 – Postnatal care up to 8 weeks after birth [9]
**AAP and AAO**	• 2016 recommendations [10] • Pediatric Eye Evaluation Preferred Practice Pattern [11]
**RCPCH**	• Identification of visual impairments (Book chapter with recommendations) [12]
**UK NSC**	• 2013 recommendations [13] • Childhood vision screening. External review against programme appraisal criteria for the UK National Screening Committee 2019 [14]
**Systematic reviews**	
**Cochrane Library**	• Antonio-Santos 2014 – Occlusion for stimulus deprivation amblyopia (Review) [15] • Hull 2017 – Tests for detecting strabismus in children aged 1 to 6 years in the community (Review) [16] • Korah 2014 – Strabismus surgery before versus after completion of amblyopia therapy in children (Review) [17] • Powell 2009 – Vision screening for amblyopia in childhood (Review) [18] • Tailor 2015 – Binocular versus standard occlusion or blurring treatment for unilateral amblyopia in children aged three to eight years (Review) [19] • Taylor 2014 – Interventions for strabismic amblyopia (Review) [20] • Taylor 2012 – Interventions for unilateral and bilateral refractive amblyopia (Review) [21]

**Abbreviations:***AAO* American Academy of Ophthalmology, *AAP* American Academy of Pediatrics, *CDC* Centers for Disease Control and Prevention, *NICE* National Institute for Health and Care Excellence, *PrevInfad* PrevInfad workgroup from the Spanish Association of Primary Care Pediatrics, *RCPCH* Royal College of Paediatrics and Child Health, *UK NSC* UK National Screening Committee, *USPSTF* US Preventive Services Task Force, *WHO* World Health Organization

## Existing recommendations

We summarized the existing recommendations and the strength of recommendations as per their authors in Table 2.

**Table 2:** Summary of existing recommendations

Source	Ref	Date	General recommendations for vision screening in children^**a**^		
			Newborns and infants < 6 months	Children 6 months to 3 years of age	Children 3–5 years of age
**USPSTF**	[4]	2017	–	“The USPSTF concludes that the current evidence is insufficient to assess the balance of benefits and harms of vision screening in children younger than 3 years.” (***I Statement***)	“The USPSTF recommends vision screening at least once in all children aged 3 to 5 years to detect amblyopia or its risk factors.” (***Grade B recommendation***)
**PrevInfad**	[5]	2016	“PrevInfad recommends ocular inspection and red reflex test in each well-child visit on the first six months of life, considering that, although the quality of evidence is poor, the expected balance of the intervention is probably positive. (Grade of recommendation: given that the red reflex test does not meet the necessary conditions to be considered a screening test, it is not possible to establish a grade of recommendation for the newborns and infants’ visual disorders screening.)”	–	“PrevInfad recommends visual disorders screening (amblyopia, strabismus and refraction errors) at the age of 3 to 5 years old.” (***Grade B recommendation***)
**CDC ‘Prevent blindness’**	[8]	2019	Newborns should have their eyes checked while still in the hospital nursery. This examination in the nursery should be for general eye health and include a red reflex test. This examination can help detect several congenital eye problems, some of which can be very serious and permanently threaten vision.	*During regular well baby exams, from birth to 3 years of age:* Pediatricians should use family vision history and a vision assessment to see if vision problems exist.	Beginning at well child exams at age 3 and continuing annually through 10 years of age, vision screenings should be performed assessing your child’s visual acuity and ocular alignment
				*If a child fails a vision screening or there is any concern of an eye or vision problem:* The child should be referred for a comprehensive professional eye examination. This combination of vision screenings with referral for a comprehensive professional eye examination are the recommendations of the American Academy of Pediatrics, the American Academy of Ophthalmology, and the American Association for Pediatric Ophthalmology and Strabismus. The American Optometric Association supports a comprehensive professional eye examination performed by an eye doctor at age 6 months, 3 years and 5 years for all children.	
**NICE**	[9]	2015	“Appropriate recommendations made by the UK National Screening Committee should also be carried out. A physical examination should also be carried out. This should include checking the baby’s: […] eyes; check opacities and red reflex […].”	–	–
**AAP and AAO**^**b**^	[10, 11]	2016	“Examination of the eyes and visual system should begin in the nursery and continue throughout both childhood and adolescence during routine well-child visits in the medical home.”	- At 6 and 12 months: ocular history, external inspection of lids and eyes, red reflex testing, pupil examination, ocular motility assessment (12 months), and visual acuity fixate and follow response. - From 1 to 3 years of age: all the above and instrument-based screening when available. “Visual acuity screening may be attempted in cooperative 3-year-old child”	- From 4 years of age: visual acuity and all the above (prior age group).
**RCPCH**	[12]	2019	“Examination of all babies’ eyes for the red reflex should take place in the newborn examination.” (***Strong evidence***)^c^	–	“Assess visual acuity in all 4–5-year olds as recommended by the UK National Screening Committee.” (***Strong evidence***)^c^ “Use evidence-based tests as part of the 4–5-year-old screening programme.” (***Strong evidence***)^c^
			“Alert parents to the signs of visual dysfunction, per the Personal Child Health Record.” (***Good practice***)^c^ “Carry out specialist eye examinations at appropriate intervals in high-risk groups.” (***Strong evidence***)^c^		
**UK NSC**	[13]	2013, updated in 2019	–	–	“Screening of children’s eyes should continue to be offered to all children aged 4–5 years.”

**Abbreviations:***AAO* American Academy of Ophthalmology, *AAP* American Academy of Pediatrics, *CDC* Centers for Disease Control and Prevention, *NICE* National Institute for Health and Care Excellence, *PrevInfad* PrevInfad workgroup from the Spanish Association of Primary Care Pediatrics, *RCPCH* Royal College of Paediatrics and Child Health, *UK NSC* UK National Screening Committee, *USPSTF* US Preventive Services Task Force

^a^The definitions of the grades to describe the strength of the recommendations are reported in (S2)

^b^Although the AAP and AAO did not report the strength of recommendations, authors specified that the recommendations were based on panel consensus

^c^The RCPCH rates the strength of the evidence as ‘strong’, ‘moderately strong’, and ‘emerging’. Strong evidence is defined as evidence that is ‘based on evaluations that are sufficiently rigorous to determine whether an intervention can be causally linked to improvements in outcomes. A reliable comparison group is needed, which is why randomized controlled trials are an important method of estimating impact’

## Existing evidence

The USPSTF 2017 recommendations result from a systematic review that the USPSTF commissioned in order to update their 2011 recommendations on screening for amblyopia and its risk factors in children [6]. Overall, 40 studies reported in 46 manuscripts were included, addressing one or several of the authors’ key questions on screening test accuracy, and benefits and harms of vision screening in children, and of treatment of amblyopia, its risk factors, and refractive error. For each key question, authors assessed the quality of each included study (good, fair or poor, although poor studies were excluded) and the overall strength of the body of evidence (high, moderate, low or insufficient) mentioned in the text as ‘strength of the evidence’ as per the review authors.

The supportive document developed from PrevInfad consists on a comprehensive summary of systematic reviews addressing these topics [5]. PrevInfad addressed visual screening for newborns and infants, and for children aged between 12 months and 5 years, while the systematic review conducted by Jonas et al. included children between 6 months and 5 years of age. In the present document, we decided to first address screening in newborns and small infants to address congenital cataracts and retinoblastoma, and then screening in children between 6 months and 5 years of age in order to be inclusive. It should be noted that the 6 or 12 months threshold is very unlikely to make any difference on the inclusion of the studies as there is very little evidence on this age group.

### Visual disorders screening in newborns and small infants

Congenital cataract and retinoblastoma cause opacities of the transparent media, which can be detected by a simple and safe ocular examination assessing the red reflex with an ophthalmoscope. Although there is little evidence supporting the validity of this examination or the effectiveness of screening both diseases, it is widely accepted to routinely search in the neonatal period for opacities of the transparent media or leukocoria, as both disorders can be treated [5, 12].

From several national studies, screening newborns within the first six weeks of life detected between 47% (in the UK) and 75% (in Sweden) of newborns with congenital cataract that needed surgery. It seems that the sensitivity of the red reflex to detect retinoblastoma is low, however the use of this low sensitivity screening tool is accepted in this case as it is a potentially lethal disorder and there is no alternative screening test as of today [5]. In addition, early diagnosis for early treatment is essential in both disorders. In the case of congenital cataract, surgery should be conducted within the first three months of age with the aim to achieve optimum outcome. Delay in the treatment can lead to irreversible amblyopia and consequent vision loss of the affected eye. In the case of retinoblastoma, it remains curable if treated within the first three to six months after the detection of leukocoria. Therefore, a child with leukocoria should be referred immediately. With early diagnosis and adequate treatment, the cure rate is up to 98% mostly with preservation of the vision, while survival will decrease with delay in the treatment [5].

One study was found that assessed cost-effectiveness of cataract screening in newborns. This study, conducted in Sweden, found that this screening performed in maternity wards after birth and during the routine health visits of the infant was cost-effective when compared to the screening performed only after birth [5].

### Accuracy of screening tests for detecting strabismus, refractive errors, anisometropia, and amblyopia in children under five years of age

We summarized the main tests used for detecting strabismus, refractive errors, anisometropia and amblyopia in Table 3. Three systematic reviews addressed accuracy of these screening tests, which are summarized in Tables 4 and 5.

**Table 3:** Available screening tests for visual impairment [4, 5]

Visual impairment	Type of tests	Screening tests
**Visual acuity**	Optotypes	Picture identification tests HOTV eye test Snellen Tumbling E
**Strabismus**	Ocular alignment tests	Hirschberg test (corneal light reflex test) Cover-uncover test (cross cover test) Bruckner test (red reflex test)
**Anisometropia**	Stereoacuity test	Stereo Smile Random Dot E TNO
**Refractive errors**	Instrument-based screening	Photorefraction Autorefractive screening (computerized systems that allow objective assessment of refractive errors)
**Multiple**	Instrument-based screening	Photo-screening (digitalized image)

**Table 4:** Systematic reviews on accuracy of vision screening tests

Systematic review	Objective	Methods		Number of manuscripts included
		Date of literature search	Inclusion criteria	
Powell 2009 [18]	To evaluate the effectiveness of vision screening in reducing the prevalence of amblyopia	August 2008	Randomised controlled trials (RCTs) or cluster RCTs comparing the prevalence of amblyopia in screened versus unscreened populations 12 months from screening	None
Hull 2017 [16]	To assess and compare the accuracy of tests, alone or in combination, for detection of strabismus in children aged 1 to 6 years, in a community setting by non-expert screeners or primary care professionals to inform healthcare commissioners setting up childhood screening programmes	January 2017	Prospective and retrospective population-based studies evaluating diagnostic test accuracy in consecutive participants	1 study, (Arthur 2009), which looked at the effectiveness of automated photo-screener for the detection of strabismus [22]. This study was also included in Jonas 2017.
Jonas 2017 [6]	To assess the accuracy of the several screening tests	June 2016	- Children aged 6 months to 5 years - Studies conducted in countries categorized as “very high” on the United Nations Human Development Index - Publication in English language - Good or fair quality of studies	34 studies, conducted in Europe (7 studies), the United States (19 studies), Canada (5 studies) and Australia or New Zealand (3 studies). The strength of the evidence rated by the review authors based on the 34 included studies (45,588 observations) was low.

**Table 5:** Main findings on accuracy of vision screening tests

Screening tests for visual impairment	Included studies	Main findings
**Visual acuity tests** (assessment of picture identification tests (LEA Symbols chart) and HOTV eye test)	6 studies [6]	When screening test cut-offs were set to achieve specificities of 90%: • Positive LR: 6.1 (95% CI 4.8 to 7.6); ‘an abnormal result moderately increased the likelihood of amblyopia, amblyopia risk factors (strabismus, astigmatism, hyperopia, myopia, anisometropia), or significant nonamblyogenic refractive error’ • Negative LR: 0.43 (95% CI 0.38 to 0.50); ‘a normal result indicated a small decrease in the likelihood’
**Ocular alignment tests** (Cover-uncover test)	1 study [6] (*n* = 3121)	Sensitivity to detect strabismus was 60% for specificity set to 90% • Positive LR: 7.9 (95% CI 4.6 to 14.0) • Negative LR: 0.73 (95% CI 0.15 to 0.85)
	1 study [5]	For detecting strabismus in children at 37 months: • Sensitivity of 75% (95% CI: 57.7 to 89.9%) • Specificity of 100%
**Stereoacuity tests**	4 studies [6] (*n* = 7801)	• Positive LRs: range from 3.6 to 4.9 • Negative LRs: in the minimal range for detecting amblyopia risk factors or significant nonamblyogenic refractive error and in the moderate range for detecting refractive error or strabismus
**Combination of clinical tests** (visual acuity, ocular alignment and stereoacuity tests)	4 studies [4–6] (*n* = 1854)	• Positive LRs: median of 14; range from 12 to 17 (3 studies); 4.8 (95% CI 2.8 to 8.4; 1 study; *n* = 141) • Negative LRs: median of 0.28; range from 0.10 to 0.91 • In one study, the cover-uncover test performed by professionals or a stereoacuity test showed an increased detection of strabismus when combined with visual acuity tests.
**Autorefractors**	16 studies [6] (*n* = 16,712) (5 studies recruited children < 3 years of age)	• Positive LRs: ‘most studies reported moderate positive LRs’, ‘some studies reported large positive LRs’ • Negative LRs: ‘most studies reported small negative LRs’, ‘some studies reported large negative LRs’
**Photo-screeners**	11 studies [6] (*n* = 6187)	• Positive LRs: ‘most studies reported moderate positive LRs’ • Negative LRs: ‘most studies reported small negative LRs’
	1 study [16]	Prospective study that evaluated the diagnostic accuracy of a digital photo-screener in a school screening programme in Canada [16, 22]. Among the 335 recruited children (98% were 4 or 5 years of age), 271 completed both the screening test and the reference standard test (ophthalmic examination by a physician who was blinded to the photo-screening findings). Results from both tests agreed in 94% of cases. • Sensitivity and specificity in detecting amblyopia risk factors were 83 and 95% respectively • Positive and negative predictive values were 73 and 97%, respectively. When looking more specifically at detection of strabismus, sensitivity and specificity were 46 and 97% respectively, with 13 children detected by reference standard test. The estimated prevalence of strabismus in the population was 4.8%.
**Retinal birefringence scanning** (assessment of the Pediatric Vision Scanner (REBIScan))	1 study [6] (*n* = 102)	• Positive LR: 10.4 (95% CI 5.6 to 19.4) • Negative LR: 0.0

**Abbreviations:***CI* confidence interval, *LR* likelihood ratio

#### Influence of age in accuracy and testability of screening tests

The screening of low age children could lead to an increased rate of false positives. In addition, the youngest might present less ability to complete the screening test, known as testability [5]. Jonas et al. included five studies that looked at the variation of the accuracy of visual acuity tests, a combination of clinical tests, an autorefractor and two photo-screeners due to age [4, 6]. They found that children under the age of three were not included in most of the studies. They reached the conclusion that ‘overall, data were limited and estimates were somewhat imprecise, but studies did not find any clear differences in test accuracy when results were stratified by age.’

Although testability is reported in many studies included in the Jonas et al. review, few of them reported their findings stratified by age, or for children under three years of age [4, 6]. Testability was over 90% in most of the studies, and under 80% in few studies (all the studies with testability under 80% included children under three years of age). Higher testability rates were found in older children, and testability rates for visual acuity and stereoacuity tests were lower among children under the age of three. From the VIP study, testability of Lea and HOTV optotypes was over 95% among children between 3 and 5 years, and testability of the Random Dot E was 86 and 93% among 3 and 5-year-old children respectively. For autorefractors and photo-screeners, testability rate were close to 100% among children aged 3 years or above [4–6].

### Potential harms of screening children between six months and five years of age for strabismus, refractive errors, anisometropia and amblyopia

False-positive findings in visual screening would lead to unnecessary referrals, and the associated anxiety and costs for the families and the health system. Jonas et al. reported the false-positive rates calculated from 16 studies [6]. They found false-positive rates above 75% in studies with a low prevalence of vision abnormalities (under 10%), and between 5 and 39% in populations with a high prevalence of vision abnormalities (above 20%) [4–6].

One controlled study identified by Jonas et al. evaluated the potential psychosocial effects of visual screening [23]. This prospective study compared bullying victimization (≥4 times a month) by the age of 8 among children who were patched between two groups of schools: one group that received preschool screening for amblyopia and one group that did not. Among the included 4473 children, 122 were patched. The reduction in bullying victimization among children who were offered preschool screening compared to the control group was almost 50% (25.7% versus 47.1%, with an AOR 0.39 [95%CI 0.16 to 0.92], adjusted for sex, paternal socioeconomic class, highest level of maternal education, and type of housing). Early screening leading to early intervention was associated with a decreased risk of bullying among patched children, probably because children are younger by the time they can remove the patch.

Based on the 16 observational studies and the cohort study included in the Jonas et al. review (14,196 observations), the strength of the evidence was rated as low for bullying, moderate for false-positive rates, and insufficient for other harms.

No studies were found that assessed possible harms derived from wearing glasses when unnecessary (long term outcomes and impacts in the vision), nor that evaluated rate of children who received unnecessary treatment for amblyopia or any of its risk factors [4, 5].

### Effectiveness of treatment of strabismus, refractive errors, anisometropia, and amblyopia in children between six months and five years of age

The success of the visual screening will partly depend on the effectiveness of the treatment to improve or correct amblyopia and its risk factors, in order to improve visual acuity, avoid permanent vision loss, and subsequently to improve school performance and functionality for a better quality of life. There are however no studies evaluating school performance or other functional outcomes, or quality of life [4]. There are several treatment strategies for treating amblyopia, including the correction of refractive errors by using glasses, with the addition or not of occlusive patches, opaque lens or eye drops to blur the better seeing eye. Several factors could influence the effectiveness of the treatment: age of the child when starting the treatment, the intensity of amblyopia, and therapeutic compliance [5].

We summarized the main findings on the effectiveness of different interventions for vision impairment in children from the USPSTF review, five Cochrane reviews, and an additional review from the UK National Screening Committee (NSC) that was identified in the PrevInfad document, in Table 6. The UK NSC review that was published in 2019 by the same authors to look at recent evidence to make evidence-based decisions on whether the 2012 recommendations from the UK NSC needed to be amended found no new evidence on these topics [13, 14].

**Table 6:** Systematic reviews on treatment effectiveness

Syste-matic review	Objective	Methods		Main findings
		Date of literature search	Inclusion criteria	
Jonas 2017 [6]	To assess effectiveness of treatment of amblyopia, its risk factors, and refractive error	June 2016	- Children aged 6 months to 5 years - Studies conducted in countries categorized as “very high” on the United Nations Human Development Index - Publication in English language - Good or fair quality of studies	- 3 studies included, 2 conducted in the UK, 1 in the US. - Comparison: 2 studies compared patching versus no patching (with continued eyeglasses in both groups if required); 1 study compared patching plus eyeglasses versus eyeglasses alone versus no intervention. - Participants: mean age of 4 to 5.2 years and ranging from 3 to 8 years. - Intervention: the duration of treatment was 5 and 12 weeks for the two studies comparing patching versus no patching with 52 weeks of follow-up in one of them, and one year of treatment for the third study comparing three arms, with 78 weeks of follow-up. - Outcome measured: improvement in visual acuity. - Findings: patching was associated with visual acuity improvement, although the effect was small. - The review authors concluded that early treatment of amblyopia in children aged between 3 and 5 years improved vision, with greater benefits among children with more severe vision impairment at baseline. - The strength of the evidence based on these three clinical trials (*n* = 417) was rated as moderate for improved visual acuity.
Taylor 2014 [20]	To establish the most effective treatment for strabismic amblyopia: - Impact of conventional occlusion therapy - Role of partial occlusion and optical penalisation	January 2014	- RCTs for the treatment of strabismic amblyopia - No age restriction - No language or date restriction	- 3 studies included (of which 1 is included in Jonas 2017), conducted in the US. Of them, 2 studies assessed the effect of supplementing the occlusion therapy with near activities. - Occlusion in addition to refractive correction with eyeglasses when needed, ‘appears to be more effective than refractive correction alone in the treatment of strabismic amblyopia’ and that ‘the benefit of combining near activities with occlusion is unproven’
Taylor 2012 [21]	To evaluate the effectiveness of spectacles, occlusion or both for the treatment of unilateral and bilateral refractive amblyopia	January 2012	- RCTs - Treatment for unilateral and bilateral refractive amblyopia by spectacles, with or without occlusion - No age restriction	- 8 studies included (of which 2 are included in Jonas 2017) - No trials included children with bilateral amblyopia. - Meta-analysis could not be performed due to heterogeneity with a lack of data for each outcome. - Overall, review authors concluded that ‘in some cases of unilateral refractive amblyopia it appears that there is a treatment benefit from refractive correction alone’ and that ‘where amblyopia persists there is evidence that adding occlusion further improves vision’
Antonio-Santos 2014 [15]	To assess the effectiveness of occlusion therapy for stimulus deprivation amblyopia	October2013	- RCTs or quasi-RCTs - Participants with unilateral stimulus deprivation amblyopia - No age restriction	- No trial was found that fulfilled the inclusion criteria.
Tailor 2015 [19]	To determine whether binocular versus standard occlusion or pharmacological blurring treatment for unilateral amblyopia in children between 3 and 8 years of age	April 2015	- RCTs - Participants between 3 and 8 years of age with unilateral amblyopia - Any type of binocular viewing intervention	- No trial was found that fulfilled the inclusion criteria.
Korah 2014 [15]	To assess strabismus surgery before versus after completion of amblyopia therapy on functional and anatomic outcomes	July 2014	- RCTs - Children < 7 years of age - Comparison of strabismus surgery before completion of amblyopia therapy with strabismus surgery after completion of amblyopia therapy	- No trial was found that fulfilled the inclusion criteria.
PrevInfad [5] UK NSC	To address treatment of vision impairment			- 19 included studies - Comparisons: • 7 studies compared different regimens of ocular occlusion with patches • 5 studies compared blurring the vision with atropine versus ocular occlusion • 1 study compared blurring the vision with atropine on a daily basis or during weekends • 6 studies assessed other interventions - Main conclusions: • There is no evidence of any specific intervention being more effective • More intensive occlusive treatment with patches were beneficial to older children and those with severe amblyopia • In children with moderate amblyopia, blurring the eye with atropine twice weekly and patching were similarly effective, and the risk of local and systemic adverse effects caused by atropine are compensated by the psychosocial effects of patching

**Abbreviation:***RCTS* randomized controlled trials

#### Influence of age in effectiveness of treatment

Evidence on the impact of age in the effectiveness of treatment is controversial, and we report here findings from the PrevInfad summary document [5]. No clinical trial included children under three years of age. Among children between 3 and 6 years of age, most of the studies showed no association between age and effects of treatment. A meta-analysis of four trials conducted in the US including 996 children between 3 and 12 years of age, looked at three age groups: 3 to less than 5 years, 5 to less than 7 years, and 7 to less than 13 years. Treatment in the oldest group was less effective than in the other two groups (*p* < 0.04 for moderate intensity amblyopia and *p* < 0.01 for severe intensity amblyopia). There were no significant differences in treatment between the two younger groups for both moderate and severe intensity amblyopia, although there was a trend to a minor effect in older children with severe amblyopia. From another study, delaying patching by one year in children aged between three and five years was associated to poorer results after six months of follow-up. Another study compared less than three hours with three to six hours of daily occlusion in two age groups. There were no significant differences between the two regiments among children under four years (*p* = 0.54), but treatment was more effective with three to six hours daily occlusion among older children (*p* = 0.03 in 4 to 6-year-old children and *p* < 0.001 in children older than 6 years).

Although it was considered that amblyopia was irreversible if left untreated until the age of 6–10 years, some studies have shown that treatment can be effective in older children, although effectiveness is less from the age of 7 [5].

### Potential harms of treating strabismus, refractive errors, anisometropia, and amblyopia in children between six months and five years of age

Potential harms derived from treatment of visual impairment were evaluated by the Jonas review and the PrevInfad document [5, 6]. Some studies have associated the treatment of amblyopia with a reversible vision loss in the non-amblyopic eye. In one study, this was higher among children treated with occlusion, compared to atropine (decrease in visual acuity of two or more lines: RR 0.93 [95% CI 0.88 to 0.97]). Another study also showed a decrease in the visual acuity of the non-amblyopic eye, which was higher among the group treated with atropine and flat lens compared to atropine alone (decrease in visual acuity of one or more lines: RR 0.86 [95% CI 0.78 to 0.95]). This loss in visual acuity was recovered in 19 out of the 20 affected children and in 17 out of the 18 affected children in the first and second study respectively. However, other studies showed no association between treatment and increased risk of vision loss in the non-amblyopic eye, such as two trials included in the Jonas review (one trial with patching and the other comparing eyeglasses plus patching versus eyeglasses alone versus no treatment) [5].

Two clinical trials included in the Jonas review assessed the psychosocial adverse effects of treatment for amblyopia. One trial found no differences on being happy, cooperative, or good tempered, on teasing, on problems at preschool, or in emotional and behavioural problems between three groups of 4-year-old children treated with patch and eyeglasses, eyeglasses alone, and no treatment. However, they found that children treated with patch and eyeglasses were more upset than those treated with eyeglasses alone (85% versus 29% at 4 years of age [*p* = 0.03], and 62% versus 26% at 5 years of age [*p* = 0.005]). The limitation of this study is the high attrition bias, with data available for only 44% of the recruited children. The other trial showed a decrease in the emotional well-being of children treated with atropine or patch, with a larger effect among patched children. Other observational studies evaluated the association between psychosocial effects and stigma and treatment of amblyopia, with diverging findings [5, 6].

Treatment with patches was associated with cutaneous irritation in 5% of children included in one trial [5]. Another trial including 60 children comparing daily 3 h of patching, 6 h of patching and no treatment showed no inverse amblyopia and no patch allergy [6]. Some local and systemic adverse effects associated with atropine were also reported in some clinical trials [5].

The strength of the evidence rated by Jonas et al. based on the three clinical trials included for this topic (*n* = 417) was low.

### Effectiveness of population-based screening in children between six months and five years of age

No RCT was found that assessed population-based screening for vision impairments in children versus no screening, looking at long-term outcomes such as amblyopia prevalence, schooling performance or quality of life [5]. The Jonas et al. review identified two studies (one RCT and one cohort study) conducted in England in 1991–92, that evaluated the prevalence of amblyopia at the age of 7.5 years [6]. The RCT recruited 3490 children and compared intensive orthoptist screening six times between 8 and 37 months and one time at 37 months of age. The risk of presenting amblyopia at 7.5 years was lower in the group with multiple times screening compared to single time screening (0.6% versus 1.8%; RR 0.35 [95% CI: 0.15 to 0.86]), however this was only significant for one of the wo amblyopia definitions evaluated (interocular difference in acuity ≥0.3 logMAR). Mean visual acuity in the worse eye after treatment (patching) was better among children screened several times versus a single time (0.15 versus 0.26 logMAR; *p* < 0.0001) [6]. The cohort study recruited 6081 children and compared single screening at 37 months of age in one health district versus no preschool screening in two other health districts. There were no significant differences in the prevalence of amblyopia at 7.5 years of age, assessed by three definitions. Both studies were at high risk of attrition bias. Indeed, data was available for only 55% of the recruited children at the end of the follow-up period [6]. In addition, the RCT was at high risk of selection bias as the method of randomization was inadequate and the baseline characteristics for amblyopia or its risk factors were not reported. Based on these two studies (*n* = 7795), the strength of the evidence was rated as low.

Few retrospective population-based studies found an association between vision screening and a reduced prevalence of amblyopia, but the low quality of methodology raises concern about the internal and external validity of these findings [5].

It is worth to mention that there is no evidence to determine the optimal vision screening interval in children under five years of age [4].

## Summary of findings

- Although there is little evidence supporting the validity and effectiveness of examining all newborns for congenital cataract and retinoblastoma through the red reflex examination, examining routinely the eyes of all newborns is widely accepted due to the severity of both diseases, if left untreated, and the good outcomes reached by early detection and treatment.
- Visual tests used for screening children aged three to five years were assessed to be useful for detecting amblyopia and its risk factors. Estimates showed higher positive likelihood ratio for the detection of children at higher risk, and for the combination of clinical tests.
- Overall, there is a moderate certainty of evidence that visual screening in children between three and five years provides a moderate net benefit, as assessed by the US Preventive Services Task Force (USPSTF): vision screening tests are accurate for detecting amblyopia and its risk factors, and treatment of these visual abnormalities is associated with visual improvement.
- Overall, there is uncertain evidence on whether screening amblyopia and its risk factors in children under three years of age provides net benefits. Among populations with a low prevalence of vision abnormalities, screening the youngest is associated with an increased rate of false positives, leading to unnecessary additional assessment.
- There is limited evidence on harms from treatment, but patching may have some psychological harms.
- There are several gaps in the evidence to acquire a better understanding of the best combination of screening tests, the optimal age for initiation of screening, the optimal screening intervals, the net benefit of screening and treatment in children under three years of age, and the long-term outcomes of preschool screening such as school performance and quality of life.

## Data Availability

Not applicable.
